# Supplementary figures and images for: Tissue- and sex-specific small RNAomes reveal sex differences in response to the environment
Source: PLoS Genet. 2019 Feb 8;15(2):e1007905. doi: 10.1371/journal.pgen.1007905 (PMC6383947; doi:10.1371/journal.pgen.1007905)

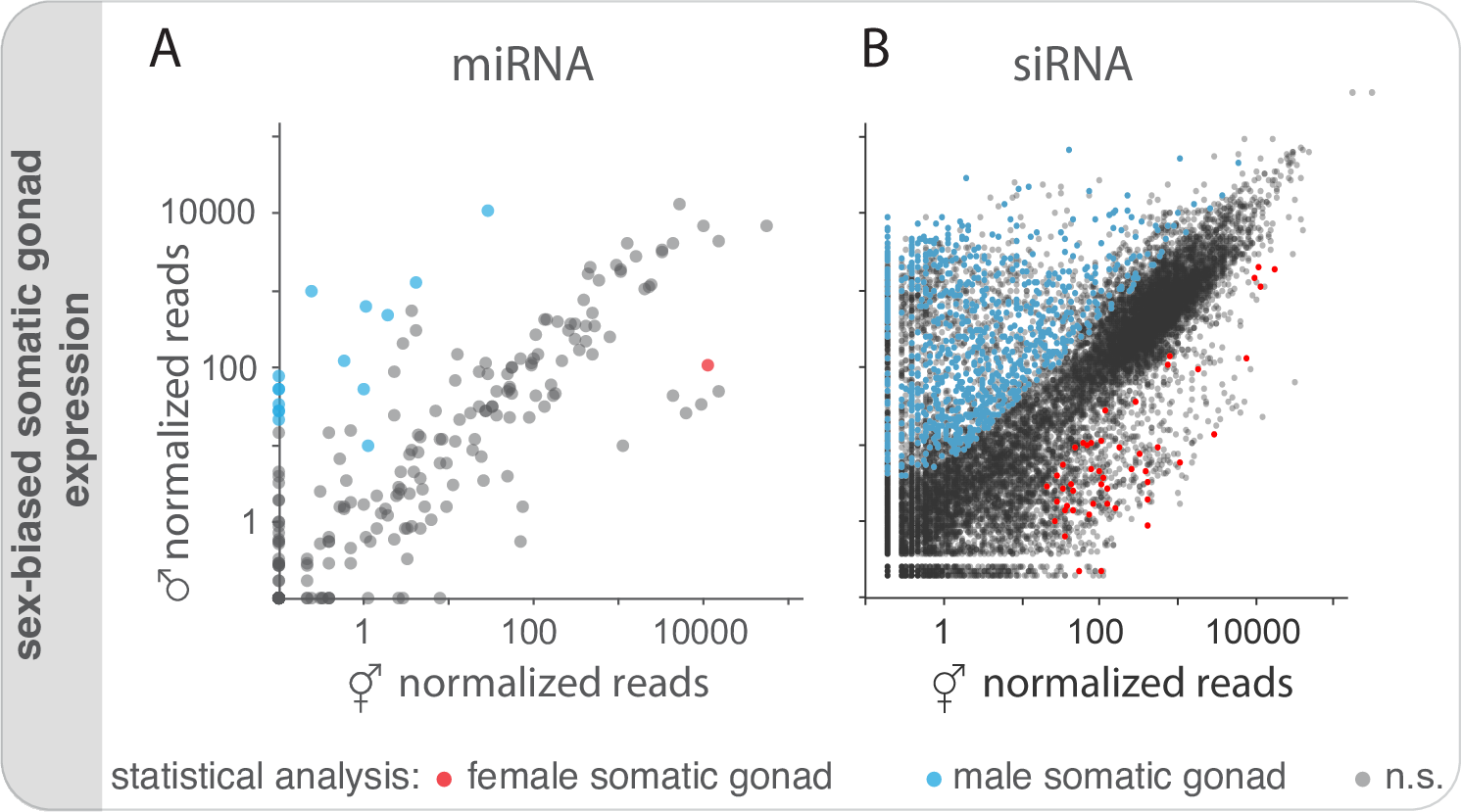

Supplement: S1 Fig — A) Mean normalized miRNA reads (sense) in hermaphrodite (n = 6 replicates) and male gonads (n = 3). miRNA expression-differences with four-fold difference in abundance that were statistically different (Wilcoxon rank sum test with continuity correction; p adjusted<0.01) between gonads of hermaphrodites and males as well as between hermaphrodites and fog-3 males (n = 2) are highlighted in red (female somatic gonad-biased) and blue (male somatic gonad-biased). Related to Fig 1D. B) Mean normalized endogenous siRNA reads (antisense) in hermaphrodite and male gonads; same analyses and representation used as in S1A Fig. Related to Fig 1H. (TIF) [file pgen.1007905.s001.tif]

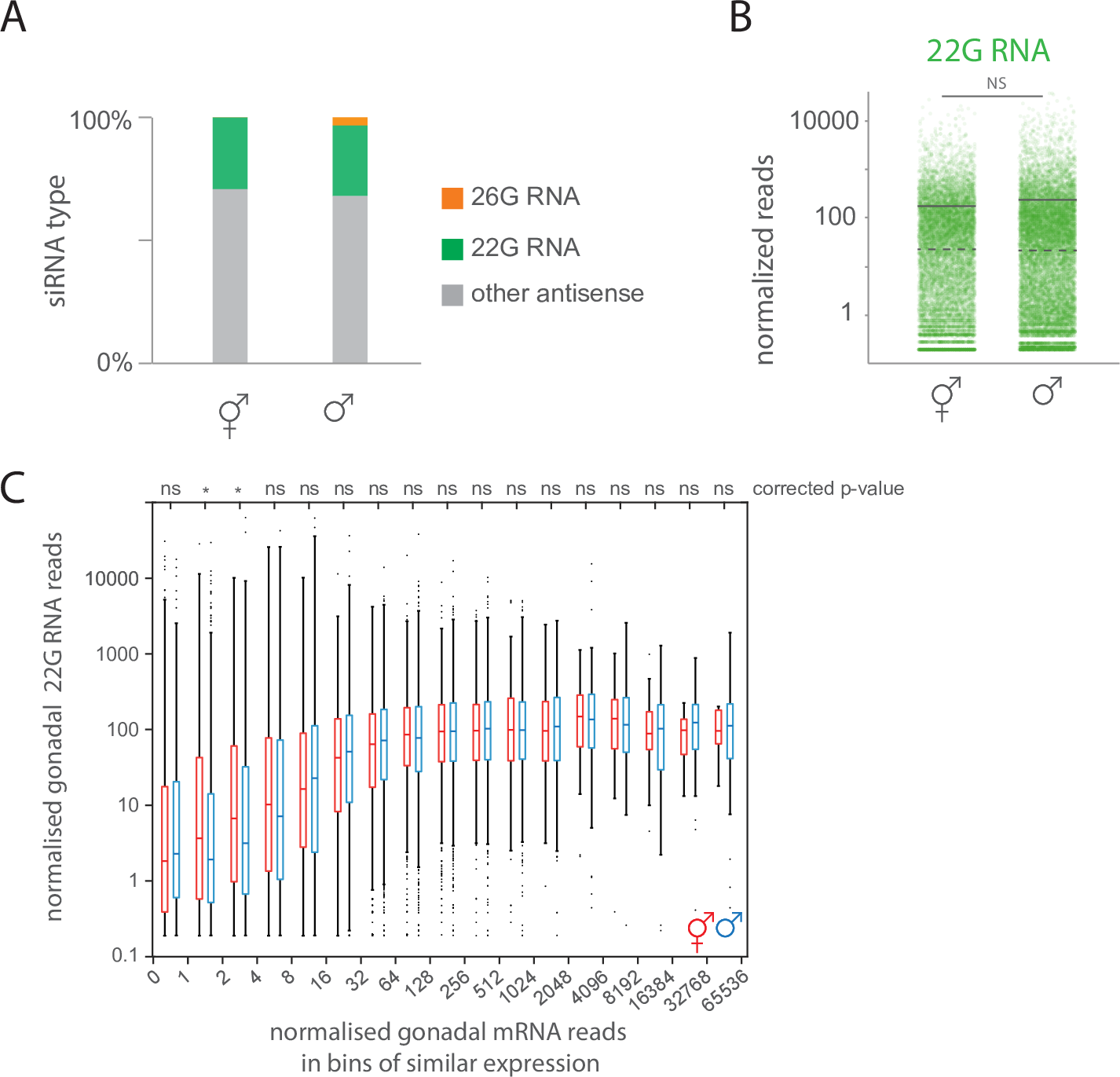

Supplement: S2 Fig — A) Percentage of antisense reads in hermaphrodite and male gonads classified as primary 26G RNA, secondary 22G RNA or other siRNA types. B) Normalized mean 22G RNA reads in hermaphrodite and male gonads per gene, median dotted line, mean full line. C) Mean 22G RNA reads targeting mRNAs of similar abundance in hermaphrodite (red) and male (blue) gonads. Boxes represent first and second quartile, 1.5 interquartile range: vertical line, mean: horizontal line, outliers: circles. Bonferroni corrected p-value of Wilcoxon rank sum test give the probability that samples from a continuous distribution have equal medians. (TIF) [file pgen.1007905.s002.tif]

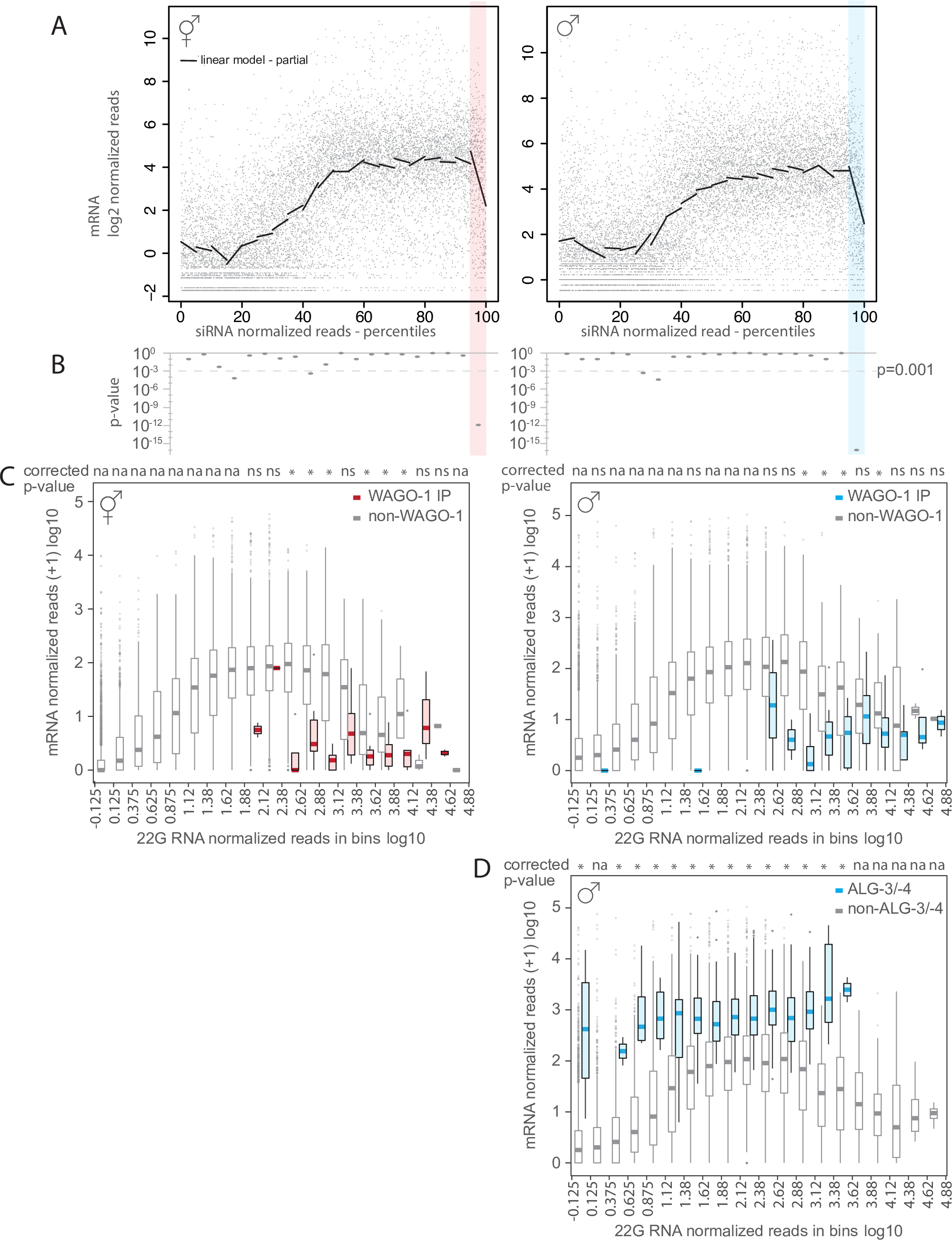

Supplement: S3 Fig — A) Mean normalized mRNA reads [55] and siRNAs in percentiles in hermaphrodite and male gonads. For 20 bins (each representing 5% of data i.e. 427 genes per bin in hermaphrodites and 504 in males) a linear model was fitted (black line). Related to Fig 2A. B) p-values indicate whether the linear model deviates from zero. The most significant negative correlation between the levels of 22G RNA and mRNA was found for the 5% of the genes with highest level of 22G RNA expression (shaded). C) mRNA expression for WAGO-1 target genes and non-target genes (Bonferroni corrected p-values of the Wilcoxon rank sum test q<0.01 indicated by stars, ns = not significant, n/a = not applicable). Data are the same as for Fig 2A but plotted as WAGO-1 targets [20] and non-WAGO-1 targets in hermaphrodites and males. Data were only available for hermaphrodites and interpolated to male samples. D) mRNA expression for ALG-3/-4 target genes and non-target genes (Bonferroni corrected p-values of the Wilcoxon rank sum test q<0.01 indicated by stars, ns = not significant, n/a = not applicable). Data are the same as for Fig 2A but plotted as ALG-3/-4 targets [34] and non-ALG-3/-4 targets in males. (TIF) [file pgen.1007905.s003.tif]

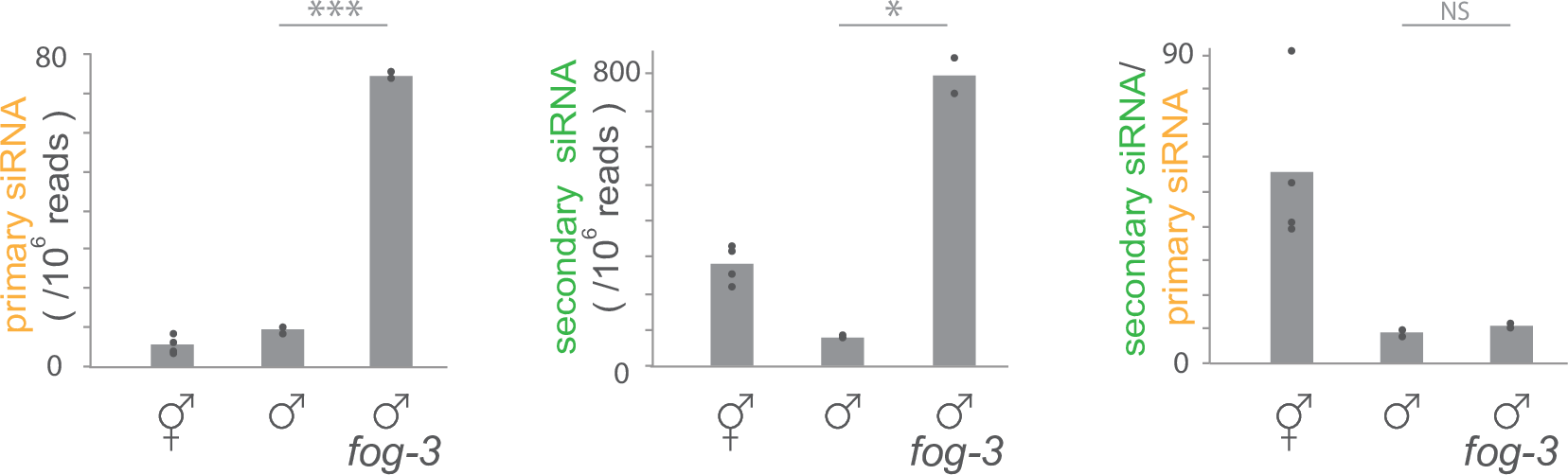

Supplement: S4 Fig — Mean primary and secondary siRNA reads normalized to total reads in gonads from fog-3 males (n = 2), control hermaphrodites (n = 4) and control males (n = 2) raised on gfp(RNAi). Data for control hermaphrodites and control males are taken from Fig 3B. The right panel shows the ratio of secondary siRNA/ primary siRNA. Means were compared with t-tests w* = p<0.05, *** = p<0.001. (TIF) [file pgen.1007905.s004.tif]

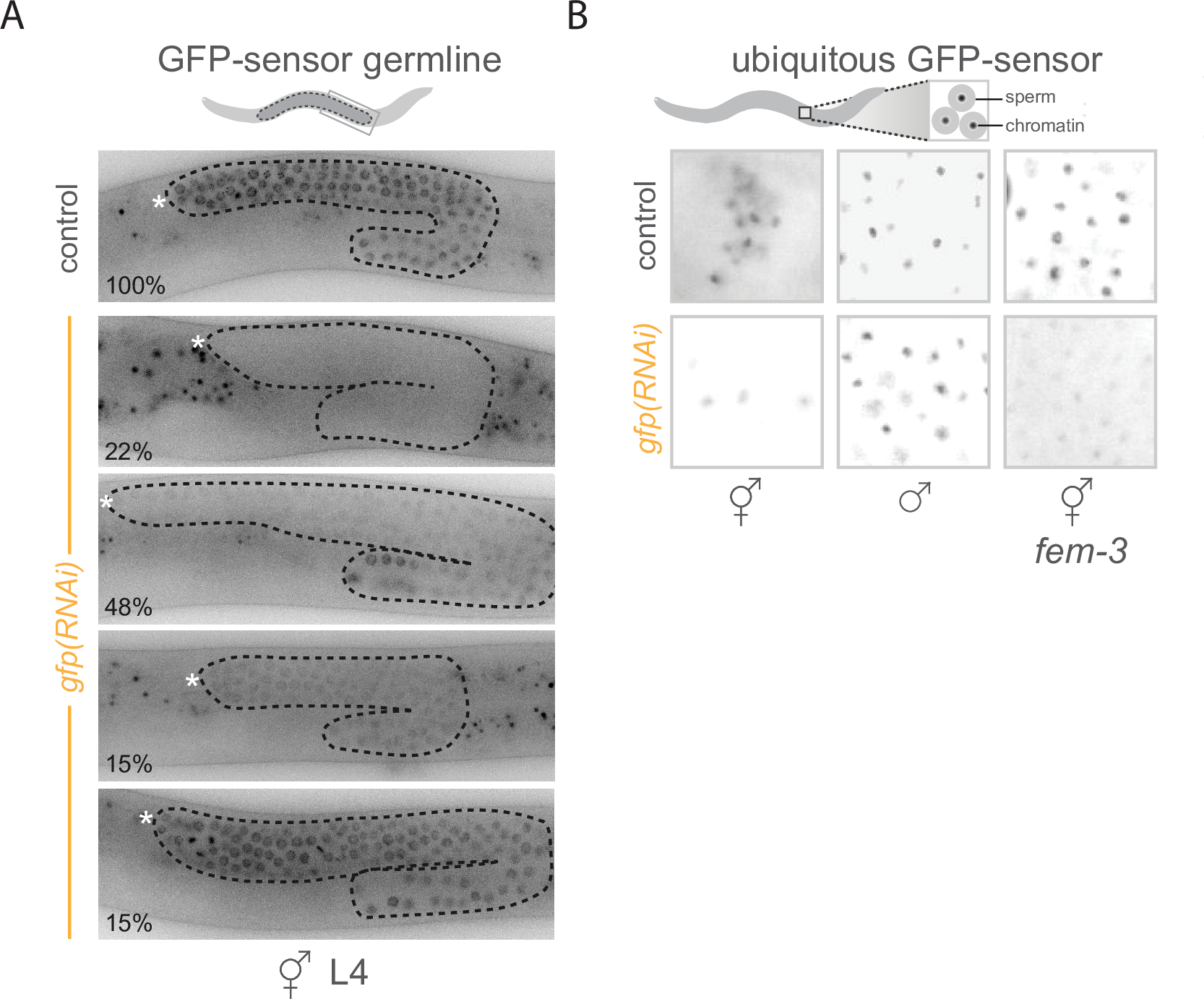

Supplement: S5 Fig — A) Fluorescence microscopy images of GFP-sensor expression of L4 hermaphrodite germlines (dotted line, distal end indicated by star); animals fed (gfp)RNAi or control RNAi. Percentage of animals with germline expression classified as: high, intermediate uniform, intermediate variable and no GFP-sensor expression (from bottom to top) for (gfp)RNAi (n = 27). 100% of animals raised on control RNAi showed high GFP-sensor expression (n = 24). B) Fluorescence microscopy images of sperm chromatin marked with a ubiquitous GFP-sensor in adult hermaphrodites, males and fem-3 hermaphrodites producing only sperm (top). Partial silencing of the GFP-sensor upon gfp(RNAi) at 24°C in control and fem-3 hermaphrodites, while control male germlines are RNAi resistant (bottom). Images were acquired with the same exposure time in control and RNAi condition, but are different between sexes due to different expression levels and physiology. For illustration purposes images are background subtracted, as explained in Fig 4D. (TIF) [file pgen.1007905.s005.tif]
